# The effect of protocol for disinfection of extracted teeth recommended by center for disease control (CDC) on microhardness of enamel and dentin

**DOI:** 10.4317/jced.52280

**Published:** 2015-12-01

**Authors:** Amin Salem-Milani, Vahid Zand, Mohammad Asghari-Jafarabadi, Parvin Zakeri-Milani, Alireza Banifatemeh

**Affiliations:** 1 DDS, MSc, Assistant Professor of Endodontics, Dental and periodontal research center, Tabriz University of Medical Sciences, Tabriz, Iran; 2 DDS, MSc, Associate Professor, Department of Endodontics, Faculty of Dentistry, Tabriz University of Medical Sciences, Tabriz, Iran; 3 PhD, Assistant Professor of Biostatistics, Department of Statistics and Epidemiology, Faculty of Health and Nutrition, Tabriz University of Medical Sciences, Tabriz, Iran; 4 PhD, Associate Professor of Pharmaceutics, Tabriz University of Medical Sciences, Tabriz, Iran; 5 DDS, Dentist, private practice

## Abstract

**Background:**

According to the guideline of the United States center for disease control (CDC), the extracted teeth should be sterilized by autoclaving or storage in 10% formalin before using for educational or research purposes. The objective of this study was to evaluate the effect of this protocol on microhardness of dentin and enamel.

**Material and Methods:**

Thirty extracted single-root teeth were used in this study. The crowns were resected, and the roots were longitudinally sectioned into two halves. The Vickers microhardness (VHN) of specimens was measured on polished canal dentin and buccal enamel surfaces. The crowns were randomly divided into three groups (n=10). Group 1 and 2 were sterilized using autoclave and formalin, respectively while group 3 (control) was stored in synthetic tissue fluid. The root halves were also randomly divided into 3 groups (n=20) which were treated as mentioned above for crown samples. Following sterilization, VHN of samples was measured again. ANOVA and paired samples t-tests were used to analyze the data.

**Results:**

Autoclaving caused a significant reduction in microhardness of dentin (*P* <0.001, 12.04% decreases in VHN). However, there were no significant differences for before and after sterilization within other groups.

**Conclusions:**

Based on the results of this study, the CDC protocol is recommended in studies related to enamel microhardness. However, Autoclaving is not an appropriate sterilization method in studies related to dentin microhardness. In these studies, two-week immersion in 10% formalin is recommended.

** Key words:**Autoclave, CDC, extracted teeth, formalin, microhardness, sterilization.

## Introduction

Human extracted teeth are commonly used in preclinical settings for education of dental students before they enter clinical environment. Blocks and manikins are other alternatives that are used in preclinic; however, they do not have the same chemical and mechanical properties of extracted teeth. Another application of extracted teeth is in laboratory researches. The new materials and techniques should be evaluated in laboratory studies prior to clinical application. Therefore, using extracted teeth remains an important part of dental education and research.

A main concern regarding the use of extracted teeth is proper infection control. Inadequate disinfection of extracted teeth makes them a potential biological hazard and source of blood-borne pathogens (1). The United States center for disease control and prevention (CDC) has developed a guideline for sterilization of extracted teeth used for research and educational purposes (2,3). According to this guideline, the teeth containing amalgam restorations should be stored in 10% formalin for two weeks before use, and the teeth without amalgam restorations should be autoclaved in 121°C/20 psi for 40 minutes (4). A sterilization procedure for extracted teeth should ideally not affect the properties of dental substrates to the extent that the “feel” and cutting characteristics are noticeably different from the clinical situation, as this is one of the major advantages in using extracted teeth for educational purposes. This is also true when the teeth are used for research purposes; because, possible alteration of chemical and mechanical properties of teeth by these methods reduces the validity of the results, and these laboratory studies will not reflect real clinical situation (5).

Different disinfectants that are used to store extracted teeth before their application in laboratory studies have been shown to influence the properties of dentin or enamel, and contradictory results of some similar studies have been attributed to the storage of extracted teeth in different storage media before study (5-8). Studies have shown that storage in 10% formalin reduces the microleakage of obturated canals, root-end fillings, and Class V composite restorations (6,9,10). However, Goodis *et al.* showed increased dentin permeability resulted from storage in formalin (11). The studies on the effect of formalin on bond strength of dentin have also shown contradictory results. Some of them showed increased bond strength (12); however, reduced or unchanged bond strength has been shown in other studies (5,7,13). The studies on the effect of autoclaving on dentinal bond strength had also contradictory results. Some revealed negative effect whereas others showed no effect on bond strength (5,7,14). To our knowledge, no study has evaluated the effect of CDC protocol on different properties of dental substrates. Therefore, there is a need for investigating the effect of sterilization procedures on different chemical and mechanical properties of teeth prior to use them for education or research purposes. One important surface property of dental substrates is surface microhardness. It is assumed that reduction in microhardness of dental hard tissues might indicate their dissolution and degradation, increasing dentin permeability, and also presenting problem for restorative procedures (15-17). Moreover, the hardness values can be related to other mechanical properties, such as Young’s modulus and yield strength (18). Therefore, the present study was carried out to evaluate the effect of CDC protocol on microhardness of dentin and enamel.

## Material and Methods

Thirty human extracted single-root teeth were used for the study. The sample size was calculated based on a pilot study (3 enamel and 6 dentin samples) with 80% statistical power. All the teeth were radiographed. The samples with caries, hypoplastic enamel or dentin, or root canal filling were excluded and replaced by intact ones. The teeth were cleaned free of attached tissue immediately after extraction using periodontal curette. The crowns were resected from CEJ using a diamond fissure bur mounted on a high-speed handpiece (NSK, Japan). The roots were longitudinally sectioned into mesial and distal halves using a diamond disk (Edenta AG, AU/SG, Switzerland). The canal surface of teeth was cleaned using a soft brush under running water. The crown and two root-halves of each specimen were fixed in autopolymerizing acrylic resin (Acropars, Marlic Medical Industries Co, Tehran, Iran) in a manner that the canal surface of roots and enamel surface of crowns were faced upwards and were not covered by acrylic resin. The surface of the samples was polished using minimum hand pressure with silicone carbide grinding papers (Buehler-Met; Agar Scientific Limited, Cambridge, UK) of 300 to 2000 grits in a progressive way under running water.

-Microhardness Test (before disinfection)

The Vickers microhardness (VHN) of specimens was measured using microhardness tester (UHL, VMHT, Walter Uhl, Germany) on polished dentin surface 1mm away from the canal and on the middle of the buccal enamel surface. The test was performed using a load of 200 grf for dentin and 500 grf for enamel samples for 20s at room temperature. The VHN for each sample was displayed on the digital readout of the tester. Each test was repeated three times, and the mean value was recorded as the VHN of each sample.

-Disinfection of the Samples

The root halves and crowns were randomly divided into following six groups:

Group 1 (Dentin, Autoclave) (n=20): The samples were autoclaved at 121°C/20 psi for 40 minutes as recommended by CDC (4).

Group 2 (Dentin, Formalin) (n=20): The samples were immersed in 10% neutral buffered formalin for two weeks as recommended by CDC (4).

Group 3 (Dentin, Control) (n=20): The samples were stored in synthetic tissue fluid (STF) for two weeks.

Group 4 (Enamel, Autoclave) (n=10): The samples were autoclaved as described for group 1.

Group 5 (Enamel, Formalin) (n=10): The samples were stored in formalin as described for group 2.

Group 6 (Enamel, Control) (n=10): The samples were stored in STF as described for group 3.

Following sterilization, VHN of each sample was measured again using the aforementioned procedures.

-Statistical Analysis

The microhardness of dentin or enamel between groups (baseline measures) was compared using one way analysis of variance (ANOVA). The measures of after intervention were compared using analysis of Covariance (ANCOVA) adjusted for baseline measures. Within group comparison to compare after and before interventions were made using paired samples t-tests. All analyses were performed using SPSS 13 (SPSS Inc., IL, Chicago, USA). *P*<0.05 was considered as statistically significant.

## Results

Regarding dentin microhardness, there observed no significant differences among groups for before or after intervention measures ( Table 1). However, there was significant differences within Autoclave group for after intervention compared with before intervention (*P*<0.001, 12.04% decreases in VHN), but this difference was not significant within Formalin or Control groups.

**Table 1:**
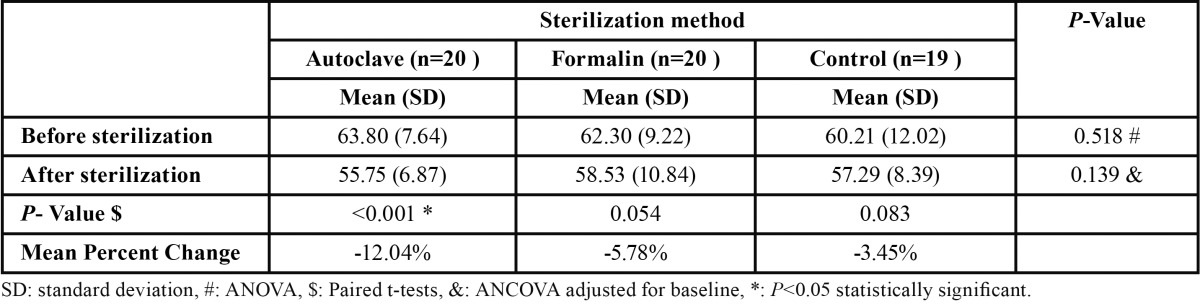
Comparison of dentin microhardness before and after intervention among study groups.

Regarding enamel microhardness, there observed no significant differences among groups for before or after intervention measures ( Table 2). Additionally, there was no significant difference within each group for after intervention compared with before intervention.

**Table 2:**
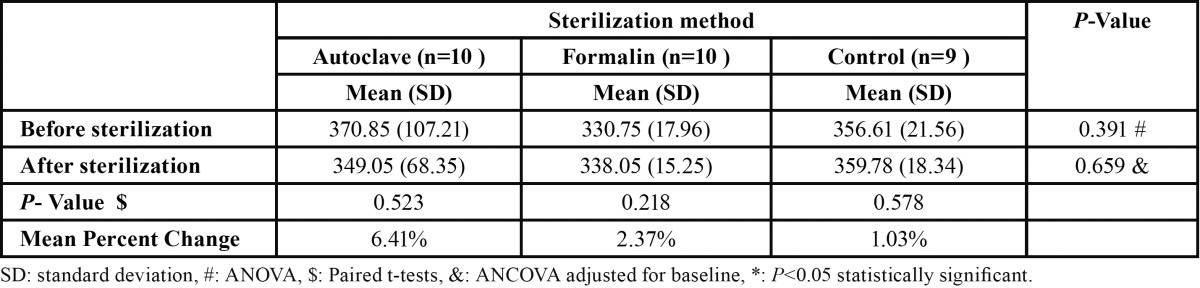
Comparison of enamel microhardness before and after intervention among study groups.

## Discussion

According to CDC protocol, the extracted teeth should be sterilized for educational or research purposes by autoclaving or immersion in 10% formalin (2,3). The purpose of this study was to evaluate the effect of this protocol on microhardness of tooth substrates. The microhardness measurement is one of the simplest non-destructive mechanical characterization methods. Vickers and Knoop hardness tests usually report approximately similar values for dental substrates (18). However, in hardness studies on tooth substrates, the Vickers test is more commonly used and recommended (18-21). Thus, this method was used in this study.

Comparison of hardness values before and after intervention was made within the same dentin or enamel sample at nearly the same place to minimize the effect of the structural variation of different teeth and variability of microhardness in different parts of a tooth (20,22).

The results of the present study showed that 10% formalin has no significant effect on microhardness of enamel or dentin. However, autoclaving reduces (12%) the microhardness of dentin.

Microhardness of dentin or enamel is believed to be dependent on the amount of mineral content in their composition (21,23,24), and its determination usually provides indirect evidence of mineral loss or gain in dental hard tissues (19,25). It is unlikely that autoclaving affects the mineral content of dentin (26). Therefore, it is not the underlying mechanism of reducing microhardness of dentin by autoclaving. Another possible explanation may be the wetting of the samples during autoclaving that may affect the hardness; however, unaltered microhardness in control samples that were immersed in PBS rules out this explanation. The exact mechanism of this reducing dentin microhardness by autoclaving needs to be found in further studies. We hypothesize that increased temperature and high pressure during autoclaving denature the organic component of dentin hence affecting the microhardness. Dentin is composed by approximately 20% wt of organic material mainly collagen fibers; however, only 4% wt of enamel is organic component (18,27). This explains the insignificant effect of autoclaving on enamel microhardness found in the present study.

CDC protocol is a useful guideline for sterilization of extracted teeth for educational or research purposes. However, its application should be limited to studies that investigate the mechanical or physical properties of dental substrates which are not influenced by sterilization procedures. As mentioned, immersion in formalin influences the microleakage (6,9,10), dentin permeability (11), and probably dentin bonding (7). Thus, it should be used in studies related to these properties with caution. However, according to the results of the present study, it does not influence dentin or enamel microhardness. Therefore, its application for sterilization of extracted teeth in researches related to dentin or enamel hardness is recommended. Autoclaving does not influence dentin permeability (28) or enamel mineralization (26). Based on the results of the present study, autoclaving the extracted teeth for researches related to enamel microhardness is also recommended. However, because of its negative effect on dentin microhardness, it is not an appropriate method of sterilization in studies that are directly or indirectly related to dentin microhardness. These studies include researches on the cutting efficacy of endodontic instruments e.g., different hand or rotary files, studies on the shaping ability or centering ability of different rotary instruments, or researches on the effect of instruments on root canal geometry.

## Conclusions

1. The CDC protocol is recommended for sterilization of extracted teeth with or without amalgam restorations in studies related to enamel microhardness.

2. Autoclaving is not an appropriate sterilization method in studies related to dentin microhardness. In these studies, two week immersion in 10% formalin is recommended for sterilization of extracted teeth.
